# Apoptotic Damage of Pancreatic Ductal Epithelia by Alcohol and Its Rescue by an Antioxidant

**DOI:** 10.1371/journal.pone.0081893

**Published:** 2013-11-14

**Authors:** Jong Bae Seo, G. A. Nagana Gowda, Duk-Su Koh

**Affiliations:** 1 Department of Physiology and Biophysics, University of Washington, Seattle, Washington, United States of America; 2 Northwest Metabolomics Research Center, Anesthesiology and Pain Medicine, University of Washington, Seattle, Washington, United States of America; 3 Department of Physics, POSTECH, Pohang, Kyungbuk, Republic of Korea; Juntendo University School of Medicine, Japan

## Abstract

Alcohol abuse is a major cause of pancreatitis. However alcohol toxicity has not been fully elucidated in the pancreas and little is known about the effect of alcohol on pancreatic ducts. We report the molecular mechanisms of ethanol-induced damage of pancreatic duct epithelial cells (PDEC). Ethanol treatment for 1, 4, and 24 h resulted in cell death in a dose-dependent manner. The ethanol-induced cell damage was mainly apoptosis due to generation of reactive oxygen species (ROS), depolarization of mitochondrial membrane potential (MMP), and activation of caspase-3 enzyme. The antioxidant N-acetylcysteine (NAC) attenuated these cellular responses and reduced cell death significantly, suggesting a critical role for ROS. Acetaldehyde, a metabolic product of alcohol dehydrogenase, induced significant cell death, depolarization of MMP, and caspase-3 activation as ethanol and this damage was also averted by NAC. Reverse transcription-polymerase chain reaction revealed the expression of several subtypes of alcohol dehydrogenase and acetaldehyde dehydrogenase. Nuclear magnetic resonance spectroscopy data confirmed the accumulation of acetaldehyde in ethanol-treated cells, suggesting that acetaldehyde formation can contribute to alcohol toxicity in PDEC. Finally, ethanol increased the leakage of PDEC monolayer which was again attenuated by NAC. In conclusion, ethanol induces apoptosis of PDEC and thereby may contribute to the development of alcohol-induced pancreatitis.

## Introduction

Here we investigate the toxic effects of alcohol. Cells die by necrosis and apoptosis when extracellular insults are intolerable [1,2]. Necrosis occurs when cells are exposed to extreme variance from physiological conditions due to external factors such as infection, toxins, or trauma [3]. Unlike necrosis, programmed cell death by apoptosis can occur under either physiological or pathological conditions including developmental cues, activation of cell death receptors, immune reactions, and exposure to chemicals [1,2,4]. Apoptosis is mediated by both a death receptor-mediated (or extrinsic) pathway and mitochondria-mediated (or intrinsic) pathway, which converge on biochemical and morphological features of apoptosis including the activation of downstream effector caspases, the cleavage of key substrates, cell shrinkage, and membrane blebbing [1,2].

Prolonged alcohol intake at high doses induces cell damage. Alcohol cytotoxicity is well studied in a variety of tissues including liver, brain, kidney, and gastrointestinal tract [5-11]. These studies have indicated that ethanol induces apoptosis through oxidative stress, mitochondrial dysfunction, and caspase activation [9-14]. In addition, the alcohol-induced cytotoxicity is attenuated by antioxidants such as N-acetylcysteine (NAC), ascorbic acid, and flavonoids including quercetin, catechin, and, resveratrol [10,14,15]. Interestingly, antioxidants are shown to suppress apoptotic signals induced by other stimuli such as hypoxia, tumor necrosis factor-α (TNF-α), and oxidative stresses including nitric oxide, hydrogen peroxide, and superoxide [16-19]. 

Some metabolites of ethanol also were shown to be toxic to cells. In the human body, ethanol is processed through both oxidative and non-oxidative pathways to reduce toxicity [20]. In the oxidative pathway, ethanol is converted to acetaldehyde by alcohol dehydrogenase (ADH), catalase, and cytochrome P450 2E1 (CYP2E1) and then acetaldehyde is further oxidized by acetaldehyde dehydrogenase (ALDH) to acetate. Ethanol is also converted to fatty acid ethyl ester (FAEE) by FAEE synthase in nonoxidative metabolism pathway. 

Ethanol has significant effects in the pancreas, and alcohol abuse is a major contributing factor of pancreatitis [21]. The pancreas has both metabolic pathways of ethanol [22]. Harmful effects of ethanol and its metabolites, acetaldehyde and FAEE, in acinar cells have been reported [23-26]. In comparison, cytotoxic effects of alcohol on pancreatic duct epithelial cells (PDEC) have not been well defined. These cells forming pancreatic ducts are important for exocrine functions [27,28] and their malfunctioning is critically involved in induction of pancreatitis [27,29]. Therefore, we investigated alcohol toxicity in PDEC for the first time and specifically addressed (1) the type of ethanol-induced cell damage (i.e. apoptotic or necrotic) (2), critical intracellular signals, and (3) involvement of metabolites such as acetaldehyde. 

## Materials and Methods

### Ethics Statement

 PDEC and gallbladder myofibroblasts were the kind gift of Dr. Sum Lee (University of Washington, now affiliated with University of Hong Kong) and the procedures including animal euthanasia, prevention of pain, and consent of human tissue use were originally approved by the Animal Experiment Committee and Human Subject Review Committee at the University of Washington.

### Solutions and Chemicals

 Mammalian saline solution contained (in mM): 137.5 NaCl, 2.5 KCl, 1 MgCl_2_, 2 CaCl_2_, 10 glucose, 10 HEPES (pH adjusted to 7.3 with NaOH). The dyes of CM-H_2_DCFDA (5 mM) and JC-1 (10 mg/mL) were dissolved as stocks in dimethyl sulfoxide. CellTiter 96® AQ_ueous_ one solution cell proliferation kit for (3-(4,5-dimethylthiazol-2-yl)-5-(3-carboxymethoxyphenyl)-2-(4-sulfophenyl)-2H-tetrazolium (MTS) assay and cytotoxicity detection assay kit for lactate dehydrogenase (LDH) were purchased from Promega (Madison, WI) and Roche Diagnostics (Mannheim, Germany), respectively. CM-H_2_DCFDA and JC-1 were from Invitrogen (Carlsbad, CA); ethanol (absolute, 200 Proof) from Fisher Scientific (Waltham, MA); staurosporine, N-acetyl-l-cysteine (NAC), carbonyl cyanide 3-chlorophenylhydrazone (CCCP), 4-methylpyrazole (4-MP), daidzin, cimetidine, disulfiram, acetaldehyde, and fluorescein from Sigma-Aldrich (St. Louis, MO). For Western blot analysis, rabbit anti-cleaved caspase-3 antibody was purchased from Abcam (Cambridge, MA) and goat anti-actin, anti-rabbit IgG-HRP, and rabbit anti-goat IgG-HRP antibodies from Santa Cruz Biotechnology (Santa Cruz, CA). 

### Cell Culture

 Non-transformed PDEC derived from the main pancreatic duct of dog were used and cultured as described previously [30]. Briefly dissociated cells were plated on Transwell inserts (Corning Costar, Acton, MA) coated with Vitrogen (Inamed Biomaterials, Fremont, CA) over a confluent feeder layer of human gallbladder myofibroblasts. Cells were maintained at 37°C in 5% CO_2_/95% air and fed three times weekly with Eagle’s Minimum Essential Medium (EMEM) containing 10% fetal bovine serum (FBS), 2 mM L-glutamine, 20 mM HEPES, 2% penicillin/ streptomycin (PS) solution, 1% insulin-transferrin-sodium selenite media supplement from Sigma (St. Louis, MO). For subculture, the cells in a monolayer were treated with 0.25% trypsin/EDTA at 37°C for 35 min and passaged to newly coated inserts. Cells of the passage numbers 10-30 were used for this study. For single-cell experiments, cells were cultured on small Vitrogen-coated coverglass chips in a medium conditioned by the myofibroblasts. Isolated unpolarized single cells were used 1 to 2 days after plating [31]. 

Pancreatic acinar cells were isolated from adult male Sprague Dawley rats (Harlan Laboratories, Kent, WA) as described previously [32] and following the guideline of animal use of University of Washington. Briefly the pancreas was removed and minced for 5 min in Hank's balanced salt solution (HBSS) plus 0.1% bovine serum albumin and 10 mM HEPES. After washing, the pancreatic segments were incubated in 10 mL of the HBSS with additional 1.6 mg/ml collagenase type IA (Sigma) for 30 min at 37°C. The solution containing collagenase was removed and replaced with DMEM/F12 (Invitrogen) plus 2% PS, 10% FBS, and 25 ng/ml hEGF (PromoKine, Heidelberg, Germany). The suspension was filtered through a 100-μm mesh (BD Biosciences, San Jose, CA) and then allowed to equilibrate for 24 h at 37°C in 5% CO_2_/95% air prior to use.

### Cell Viability Assays

 PDEC were seeded into 96-well plates coated with Vitrogen in HBSS mixture (1:5) at a density of 5 × 10^3^ cells per well in 150 μL medium (without phenol red) conditioned by myofibroblasts. Proliferating PDEC were allowed to attach to the plate for 24 h before treatment. Subsequently the cells were treated with ethanol in the absence or presence of test compound(s). After 1, 4, or 24 h incubation, the cells were washed twice with MEM (Minimum Essential Medium without phenol red) and replaced with fresh culture medium (150 μL) including a MTS reagent (10 μL) and, then, were further incubated for 2 h without light. This MTS assay measures the reducing potential of the cell. Live cells reduce the MTS reagent to a colored formazan product. Formazan was quantified by measuring the absorbance at 490 nm and 690 nm using a SpectraMax M5 microplate reader (Molecular Devices, Sunnyvale, CA).

Cell death was assessed by measuring the LDH released into the cell culture medium. For LDH assay, the culture medium was centrifuged at 1,500 rpm for 10 min at 4 °C. The supernatants were used to measure LDH activity using a cytotoxicity LDH detection assay kit according to the manufacturer’s instructions. LDH release was estimated as relative to the maximal LDH release determined by lysing the cells with 0.5% Triton-X 100 detergent. For the colorometric LDH assay, absorbances were measured in a similar way as the MTS assay. All experiments were repeated at least three times.

### Apoptosis *versus* Necrosis Detection Assay

 PDEC were plated onto Lab-Tek™ chambered coverglasses (Thermo Scientific, Rockford, IL) coated with Vitrogen and HBSS mixture (1:5 ratio) at a density of 1.0 × 10^4^ cells per well in 400 μL culture medium. After 24 h of settling, the cells were treated with ethanol for 4 h and then stained with the Promokine apoptotic/necrotic/healthy cell detection kit (PromoCell, Heidelberg, Germany) according to the manufacturer’s instruction. At the early stage of apoptosis, phosphatidyl serine (PS) is translocated from the inner leaflet to the outer side of the plasma membrane. Since annexin V has a high affinity for PS, annexin V conjugated to fluorescein (FITC) labels apoptotic cells. The plasma membrane of necrotic cells looses its integrity in necrotic cells and allows the permeation of ethidium homodimer III that binds to DNA [33,34]. The cells stained with annexin V antibody alone or together with ethidium homodimer III were counted as early or late stages of apoptotic cells, respectively [34]. The cells labeled with ethidium homodimer III alone were counted as necrotic cells. Images were obtained using a Zeiss 710 laser-scanning confocal microscope.

### Reactive Oxygen Species (ROS) Measurement

 To monitor the ROS generations, cells were incubated with 10 μM 5-(and-6)-chloromethyl-2',7'-dichlorodihydrofluorescein diacetate (CM-H_2_DCFDA) in saline solution for 30 min at 37 °C and washed twice with dye-free saline. This cell-permeable dye accumulates in the cytoplasm and it is trapped in the cell by cleavage of the acetate groups by cellular esterases. Upon oxidation, the dye becomes fluorescent 2',7'-dichlorofluorescein (DCF). This reaction is irreversible so that DCF fluorescence increases steadily even in control condition. For convenience, we subtracted a fitted control slope so that the DCF trace remains flat in the control. The dye was excited at 475 nm and fluorescence signals were recorded at > 525 nm every 15 s using a charge-coupled device (CCD) camera (Evolve, Photometrics, Tucson, AZ). Background fluorescence measured in a cell-free region was subtracted. Image processing and data analysis were done with MetaFluor software (Molecular Devices). 

### Mitochondrial Membrane Potential (MMP) Measurement

 Cells were incubated with JC-1 dye (10 μg/mL) in saline solution for 30 min at 37 °C and washed twice. MMP was monitored by determining the relative amounts of dual emissions from mitochondrial JC-1 monomers (green) and aggregates (red). Due to low and negative MMP (around -200 mV), many dye molecules accumulate and aggregate inside the mitochondria. Depolarization of mitochondrial potential causes the release of the dye into the cytoplasm so that the ratio of red/green fluorescence intensity decreases. Green monomers were excited at 488 nm and emission was detected at 518-540 nm using the Zeiss 710 confocal microscope. Red aggregates were excited at 561 nm and its fluorescence was measured at 591-700 nm. 

### Reverse Transcription and Polymerase Chain Reaction (RT-PCR)

 In order to test expression of genes for oxidative metabolic enzymes for ethanol, total RNA was isolated from PDEC with PureLink® mini kit (Invitrogen). First-strand cDNA was synthesized by reverse transcription of 2 μg of total RNA with SuperScript® III first-strand synthesis system (Invitrogen) according to standard protocols. cDNAs were then subjected to polymerase chain reaction (PCR). Primer pair sequences for the oxidative metabolism of alcohol related genes and the expected size of PCR products are described in Table 1. PCRs were run on a PerkinElmer 2400 Geneamp PCR machine in a final volume of 20 μL containing 1 μL of the first strand cDNA, 1 unit Platinum® Pfx DNA polymerase (Invitrogen), and primers (200 nM, each). DNA amplification was consist of an initial denaturation step of 5 min at 94 °C, and 35 cycles of 30 s at 94 °C, 30 s at 55 °C, 30 s at 68 °C, and final 7 min at 68 °C. Reaction products were then separated on a 2% agarose gel, stained with ethidium bromide, and photographed.

**Table 1:** Primer sets used for RT-PCR analysis.

**Gene**	**GenBank Accession number**	**Forward primer**	**Reverse primer**	**Exp. size (bp)**
ADH4	XM_535665	GCTGCAATCAACACTGCCAA	TCCCCAACCTACTGTTGTGC	318
ADH5	NM_00125215	AGTCTGCCTTCTCGGTTGTG	AGCGCAGCTCTCATGACTTT	333
CYP2E1	NM_00100333	TGGTCCTGCATGGCTACAAG	CGGGCAGGTAGTGCAGATAG	456
CAT	AB012918	TCGAGTGGCCAACTACCAAC	GGCGAACATTGGCTGCTATG	138
ALDH1A1	XM_535494.4	TTGCTGACTCCGACTTGGAC	CCGTTCAACACTCCTCCGAA	143
ALDH1A2	XM-858787.2	GCAGACTTGGTGGAACGAGA	GATGACCCCCTGCAAATCCA	105
ALDH2	XM_848535	TTCCAAGTATGGGCTGGCTG	ACTTCCGTGTATGCCTGCAA	198
GAPDH	AB038240	CGGCCCCTCTGGGAAGATGTGG	CCTTGGCAGCGCCAGTGGAAG	80

The expected transcript size (base pair, bp) is indicated in the right column.

 Quantitative PCR (Q-PCR) was performed on MX3000P® system (Stratagene) with iTaq Universal SYBR® Green Supermix (Bio-Rad) and the results were analyzed according to the manufacturer's instructions. PCR reaction was conducted as follows; 95 °C for 3 min followed by 40 repetitive thermal cycles (95 °C for 15 s, 55 °C for 30 s, 72 °C for 20 s). Dissociation curves of PCR products confirmed specific amplification of specific genes. GAPDH was used as an internal control to calculate the expression level.

### Western Blot Analysis

 Activated caspase-3 protein was detected by Western blot analysis. Cells were lysed with Mammalian protein extraction reagent (Thermo Scientific) containing EDTA-free protease inhibitor mixture (Roche Diagnostics) and then centrifuged at 12,000 rpm for 10 min. The supernatants were separated by a 4 - 12% NuPAGE gel and electrotransferred to a polyvinylidene difluoride (PVDF) membrane. The membrane was blocked in TBST (10 mM Tris-HCl, 0.1% Tween 20, pH 7.4) containing 5% non-fat dried milk for 2 h and then incubated with rabbit anti-cleaved caspase-3 (1:500 dilution) or goat anti-actin (1:1,000 dilution) antibody at 4 °C for overnight. After a washing step, the membrane was incubated with HRP-conjugated goat anti-rabbit or rabbit anti-goat secondary antibody (1:5,000 dilution) and visualized using the ECL system (Amersham Biosciences, Piscataway, NJ) followed by autoradiography. Intensity of the bands in the autoradiograms was estimated using ImageJ software (NIH).

### Nuclear Magnetic Resonance (NMR) Spectroscopy

 PDEC were seeded into 15 mL conical tubes at a density of 1 × 10^7^ cells in 2 mL culture medium conditioned by myofibroblasts. Subsequently the cells were incubated with 750 mM ethanol with or without 30 min pretreatment with 30 μM disulfiram (ALDH1 inhibitor) and 30 μM daidzin (ALDH2 inhibitor), and the tubes were gently agitated every 5 min. After 30 min incubation at 37 °C, the cells were washed twice with cold PBS (for control PDEC) or PBS including 750 mM ethanol (for ethanol-treated PDEC). The cells were lysed with cold 0.2% Triton-X 100 in PBS or in PBS containing 750 mM ethanol for 10 min and then centrifuged at 12,000 rpm for 10 min at 4 °C. The supernatants were quickly frozen with liquid nitrogen and stored at -80 °C for NMR analysis. In sample preparation and NMR measurement, we tried to minimize evaporation of acetaldehyde because of its low boiling temperature (20.2 °C).

 NMR experiments were performed on a Bruker AVANCE III 800 MHz spectrometer equipped with a cryogenically cooled ^1^H/^13^C/^15^N triple resonance gradient probe at Northwest Metabolomics Research Center, University of Washington. 300 μL test samples were mixed with 290 μL phosphate buffer (100 mM, pH 7.4) in deuterated water and 10 μL of 66.17 μM 3-(trimethylsilyl) propionic-2,2,3,3-d_4_ acid (TSP, sodium salt) solution in deuterated water, resulting in the final concentration of 1.1 μM for TSP and the mixed solutions were transferred to 5 mm NMR tubes. The temperature of the NMR tubes was maintained at 10 °C during NMR experiments. One-dimensional ^1^H spectra were obtained using the one pulse sequence with suppression of water peak by presaturation during recycle delay. The data were acquired using 64k complex time domain points, radio-frequency pulse angle of 60° and an overall recycle delay of 9.4 s. 128 transients were co-added and the resulting data were Fourier-transformed after zero filling to 64k points and multiplying by an exponential window function using a line broadening of 0.5 Hz. The spectra were phase- and baseline-corrected and normalized to the internal reference, i.e. TSP peak. Concentration of acetaldehyde was determined using the peak area of methyl NMR peak of acetaldehyde at 2.245 ppm relative to the area of TSP peak at 0 ppm, and by taking into account the number of protons in each molecule. For the estimation of the intracellular acetaldehyde concentration, one should consider the dilution factor of about 60 times (total cell volume of about 10 μL and final sample volume 600 μL).

### Measurement of Permeability in PDEC Monolayer

 PDEC were cultured on Vitrogen-coated membranes to form tight monolayers over 3-5 days after plating [30]. PDEC monolayers were treated with ethanol in the presence or absence of NAC for 4 h and washed twice with saline solution. Then the tightness of monolayers was estimated by perfusing a saline containing 10 μM fluorescein to the luminal side and detecting the leak of the dye to the serosal side. Fluorescein at the serosal compartment was excited at 488 nm, and fluorescence emission was detected at 492-622 nm using the Zeiss 710 confocal microscope. 

### Statistical Analysis

 All numerical values in the text and figures are given as mean ± SEM. *n* and *N* denote the numbers of analyzed wells and cells (or monolayers), respectively. Statistical significance was determined by Student’s *t*-test, and P < 0.05 was considered significant.

## Results

### Ethanol Induces Cell Damage of PDEC *via* Apoptosis

**Figure 1:**
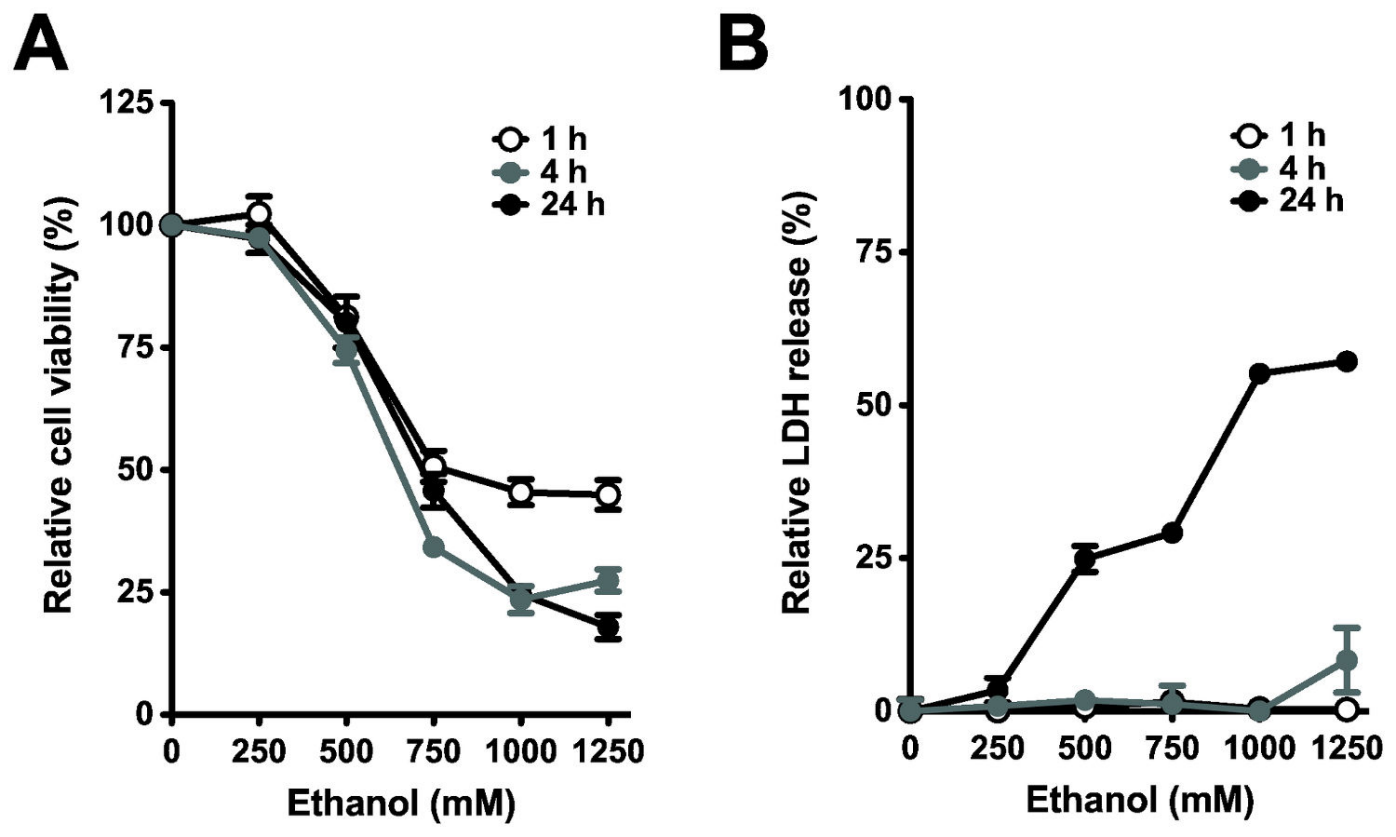
Ethanol induces cell damage in dog PDEC. (A) Cells were treated with the indicated ethanol concentrations for 1, 4, or 24 h, respectively. After the treatment, cell viability was determined with MTS assay. The values were calculated relative to the control group. The results are mean ± SEM and representative of three independent experiments (*n* = 4 - 6 for each condition). (B) After 1, 4, or 24 h exposure to the indicated ethanol, LDH activity in extracellular culture medium was measured. The values are presented as relative to LDH release by 0.5% Triton-X 100.

 We started to estimate cell damage induced by ethanol using two different assays, the MTS assay for cell viability and the LDH assay to detect necrotic release of LDH from cells. The MTS assay indicated that ethanol triggered significant cell damage in a dose- and time-dependent manner (Figure 1A). Cell viability decreased at high concentrations of ethanol (>250 mM or 1.15% volume percent) after 1 and 4 h of incubation. In contrast, release of LDH into culture medium was not significantly changed within 4 h (Figure 1B), suggesting that ethanol induces cell death via non-necrotic mechanism within 4 h incubation but then there is some cell rupture afterward, e.g. 24 h. With longer ethanol treatment (48 or 72 h), the damage was induced even at lower concentration (125 mM or 0.73%) (Figure S1A). Isolated pancreatic acinar cells from rat exhibited ethanol sensitivity comparable to PDEC, more tolerable to 4 h but more sensitive to 72 h incubation of ethanol (Figure S1B). 

**Figure 2:**
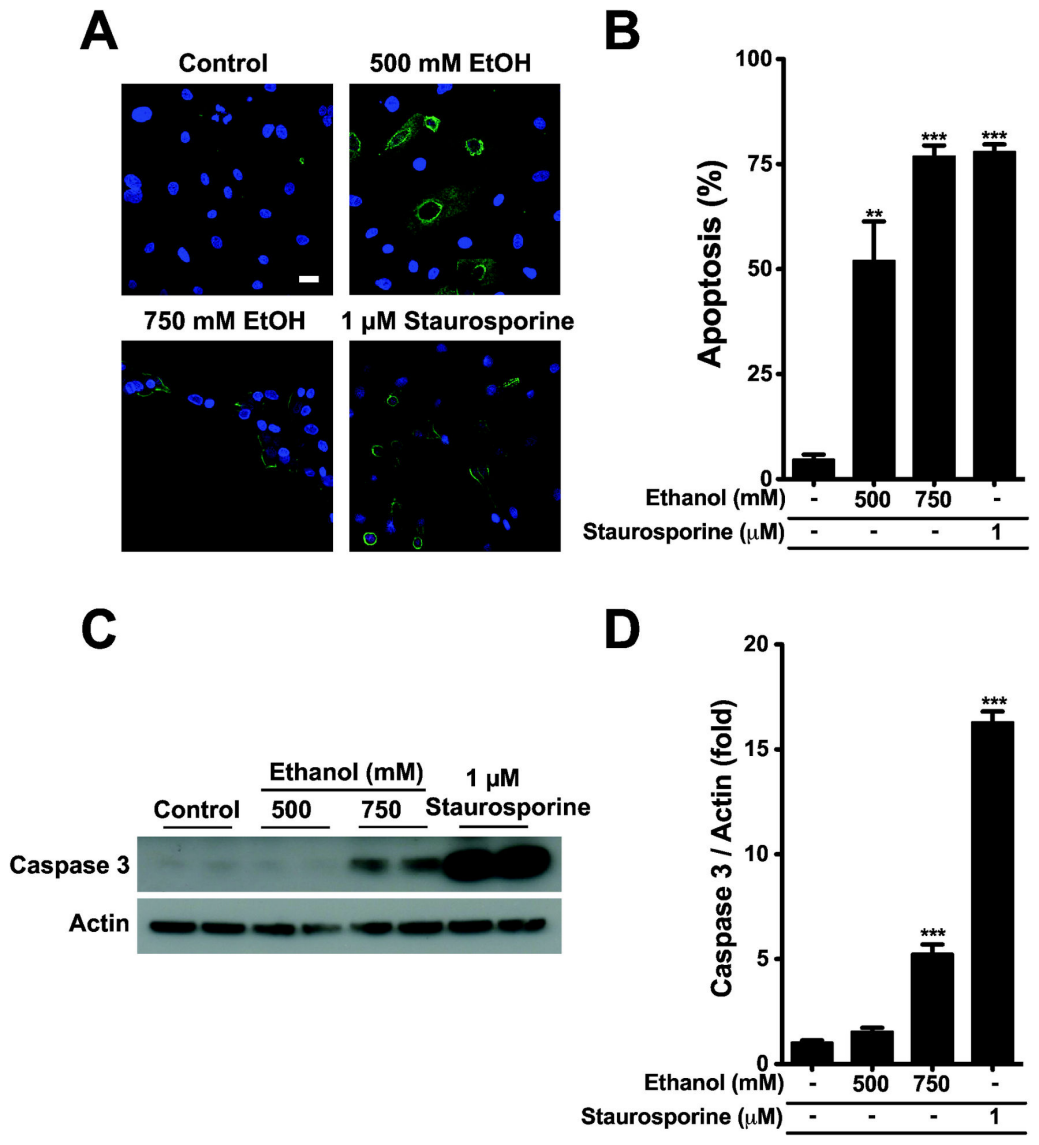
Ethanol induces apoptosis. (A) Cells were treated with the indicated ethanol concentrations for 4 h, and then stained with annexin V-FITC (green, apoptotic cells), ethidium homodimer III (red), and Hoechst 33342 (blue, nucleus staining) for 15 min. Staurosporine was a positive control to trigger apoptosis. Bar is 20 μm. (B) Percentage of apoptotic cells from two independent experiments. (C) Cells treated with 500 or 750 mM ethanol for 4 h were analyzed by Western blot to detect activated caspase-3 and actin proteins. (D) Activated caspase-3 was calibrated by actin level of the samples and presented as relative to the control. Two experiments. ** *P* < 0.01 and *** *P* < 0.001 compared to control group.

Next, we wanted to test whether ethanol-induced cell damage within 4 h is through necrosis or apoptosis using the Promokine apoptotic and necrotic cell detection assay (Figures 2A and B). Cells stained with FITC-conjugated anti-annexin V antibody alone (early apoptotic cells) or together with ethidium homodimer III (late apoptotic cells) were counted as cells dying through apoptotic pathway. Cells labeled with ethidium homodimer III alone were regarded as necrotic cells and were less than 1% of total cells in all treatment groups. Percentage of apoptotic cells after ethanol treatment was 51 ± 9 and 77 ± 3% of total cells at 500 and 750 mM, respectively. Staurosporine, a positive control, induced apoptosis in 78 ± 2% of cells. 

Finally, we assessed whether ethanol activates caspase-3, one of the major indicators of apoptosis. This assay used an antibody that specifically binds to activated caspase-3 (Figures 2C and D). The Western blot showed that 750 mM ethanol significantly stimulates caspase-3 activation by 5.2 ± 0.5 fold, suggesting that ethanol-induced damage of dog PDEC is mainly mediated *via* apoptosis. By comparison, activated caspase-3 with 500 mM ethanol was not significantly different from control, while about 50% cells were determined to be apoptotic in Figures 2A and B. This difference is probably due to the fact that conversion of PS to the outer leaflet of the membrane (apoptosis-necrosis assay) and activation of caspase-3 (Western blot) are the early and late steps of apoptosis, respectively. However, a stronger damage with 750 mM ethanol appears to accelerate the apoptosis significantly so that caspase-3 activation is evident even after 4 h of alcohol treatment. 

### Ethanol Induces ROS Generation and Mitochondrial Depolarization

**Figure 3:**
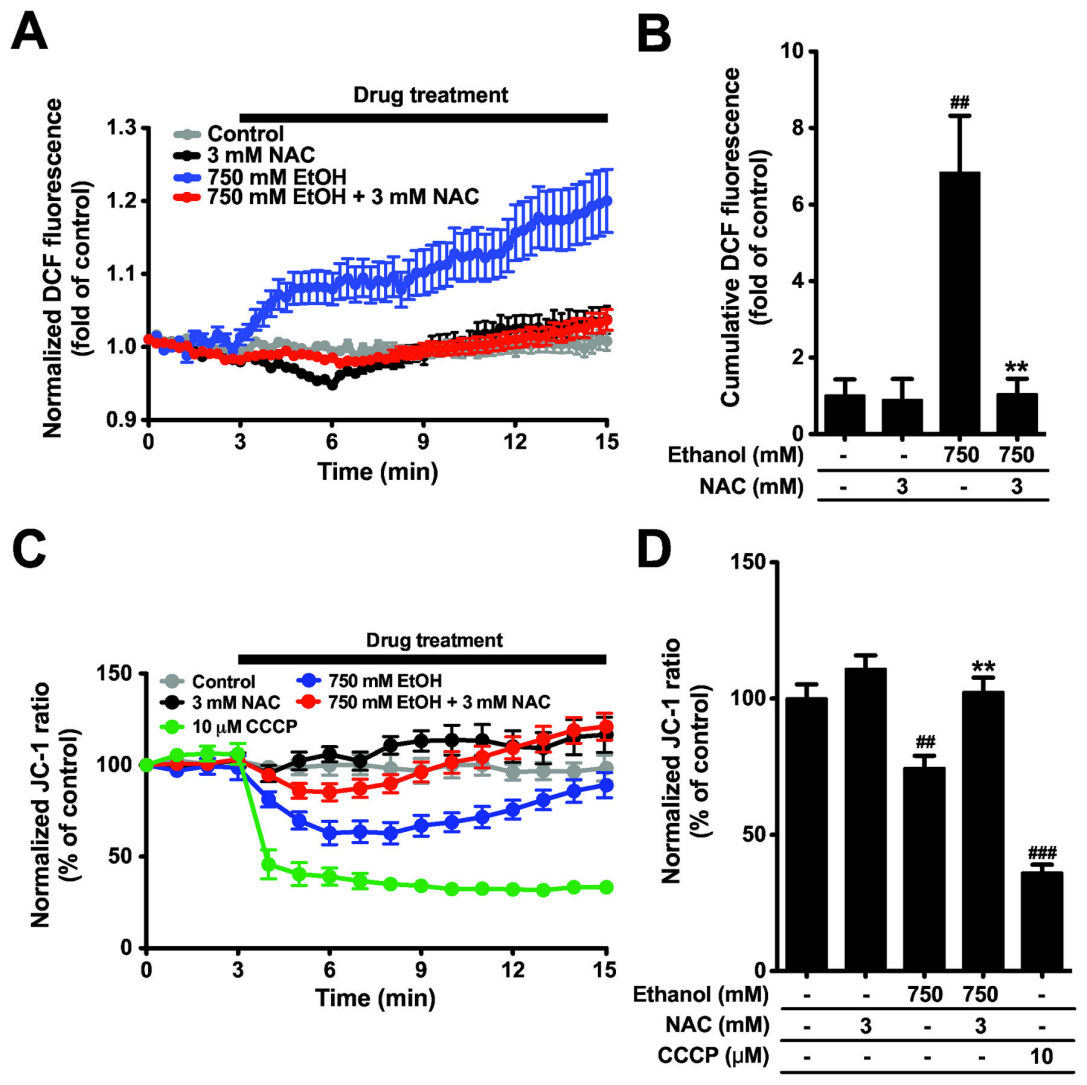
Ethanol evokes ROS generation and mitochondrial depolarization, which are blocked by an antioxidant, NAC. (A and B) ROS measured with CM-H_2_DCFDA. The areas under the curves and above the 1.0 level were calculated during drug treatments (3 to 15 min) and normalized to that of the control. (C and D) Mitochondrial membrane potential (MMP) measured with JC-1 dye. Decrease of ratio (F561/F488) indicates MMP depolarization. For the bar graph (D), MMP was evaluated as averages during drug treatments. Note that 3 mM NAC reversed ROS generation and MMP depolarization induced by ethanol. *N* = 7 - 23 for each condition, ^##^ *P* < 0.01 and ^###^ *P* < 0.001 compared to the control. ** *P* < 0.01 compared to the ethanol-treated group.

In order to address how ethanol induces apoptosis, we considered reactive oxygen species (ROS) production and mitochondria membrane potential (MMP) change. These factors are typical ways of apoptotic cell death in several cell types [10,12-14]. We used real time indicators, CM-H_2_DCFDA for ROS and JC-1 dye for MMP. When PDEC were treated with 750 mM ethanol, ROS generation was increased (blue line in Figure 3A, 6.8 ± 1.5 fold in Figure 3B) and MMP was also significantly depolarized (i.e. becoming less negative, blue line in Figure 3C, JC-1 ratio = 74 ± 5% of the control in Figure 3D). CCCP, uncoupler of oxidative phosphorylation removing proton gradient and MMP, was used as a positive control.

According to previous results, antioxidants can retard ROS generation and MMP depolarization evoked by various stimuli such as hypoxia, TNF-α, and oxidative stress [16-19]. In our cells, NAC, an antioxidant, reduced ROS generation to the control level (1.04 ± 0.41 fold of control, Figures 3A and B). Similarly, NAC abolished MMP depolarization (102 ± 5 % of control, Figures 3C and D). The protective effect of NAC implies that ROS generation is an upstream event of mitochondrial depolarization.

**Figure 4:**
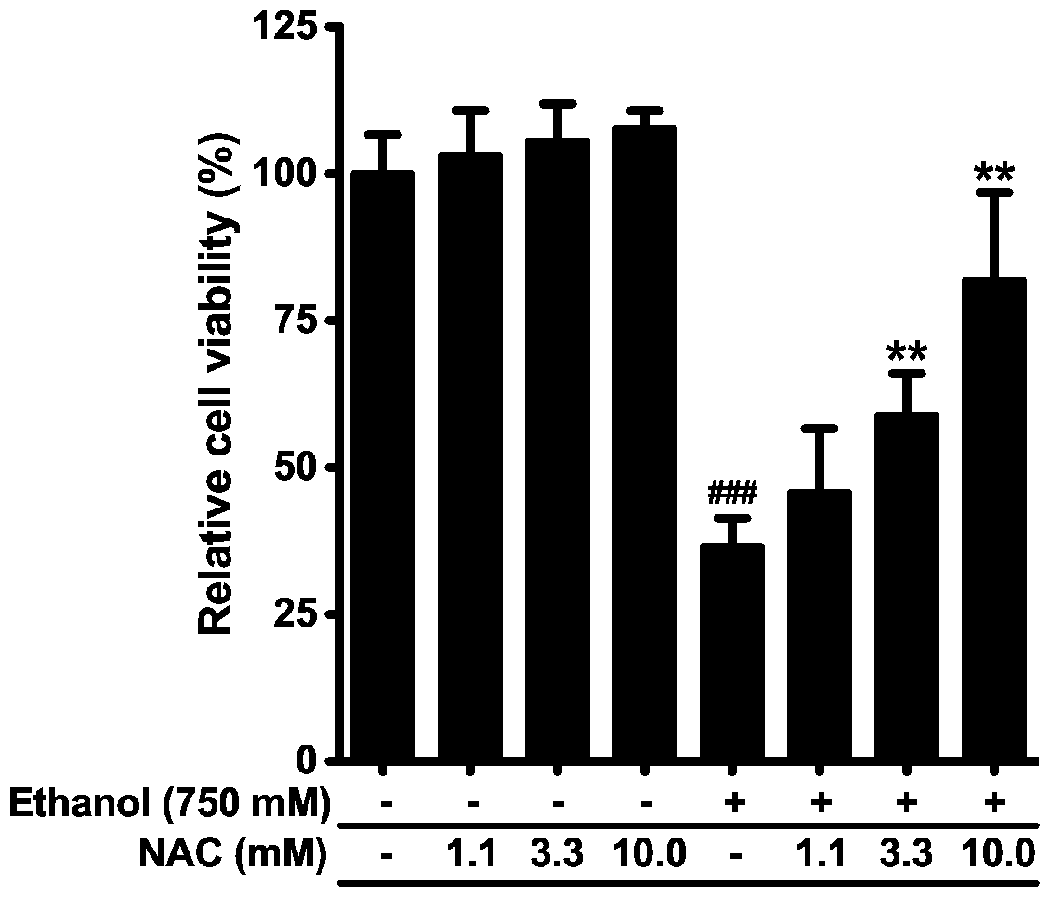
NAC reduces ethanol-induced cell damage. PDEC were treated with 750 mM ethanol for 4 h with or without 30 min pretreatment of indicated NAC concentrations, and then cell viability was measured. n = 4 - 6 for each condition, ^###^ *P* < 0.001 compared to the control. ** *P* < 0.01 compared to the cells treated with ethanol alone.

Next, we used MTS assay to address whether NAC also rescues ethanol-induced cell damage. In fact, NAC restored ethanol-induced cell damage in a dose-dependent manner (Figure 4), suggesting that ethanol-induced ROS generation and mitochondrial depolarization cause dog PDEC death. NAC alone did not have any effect on cell viability.

### Acetaldehyde as an Oxidative Metabolite of Ethanol Affects Cell Viability

**Figure 5:**
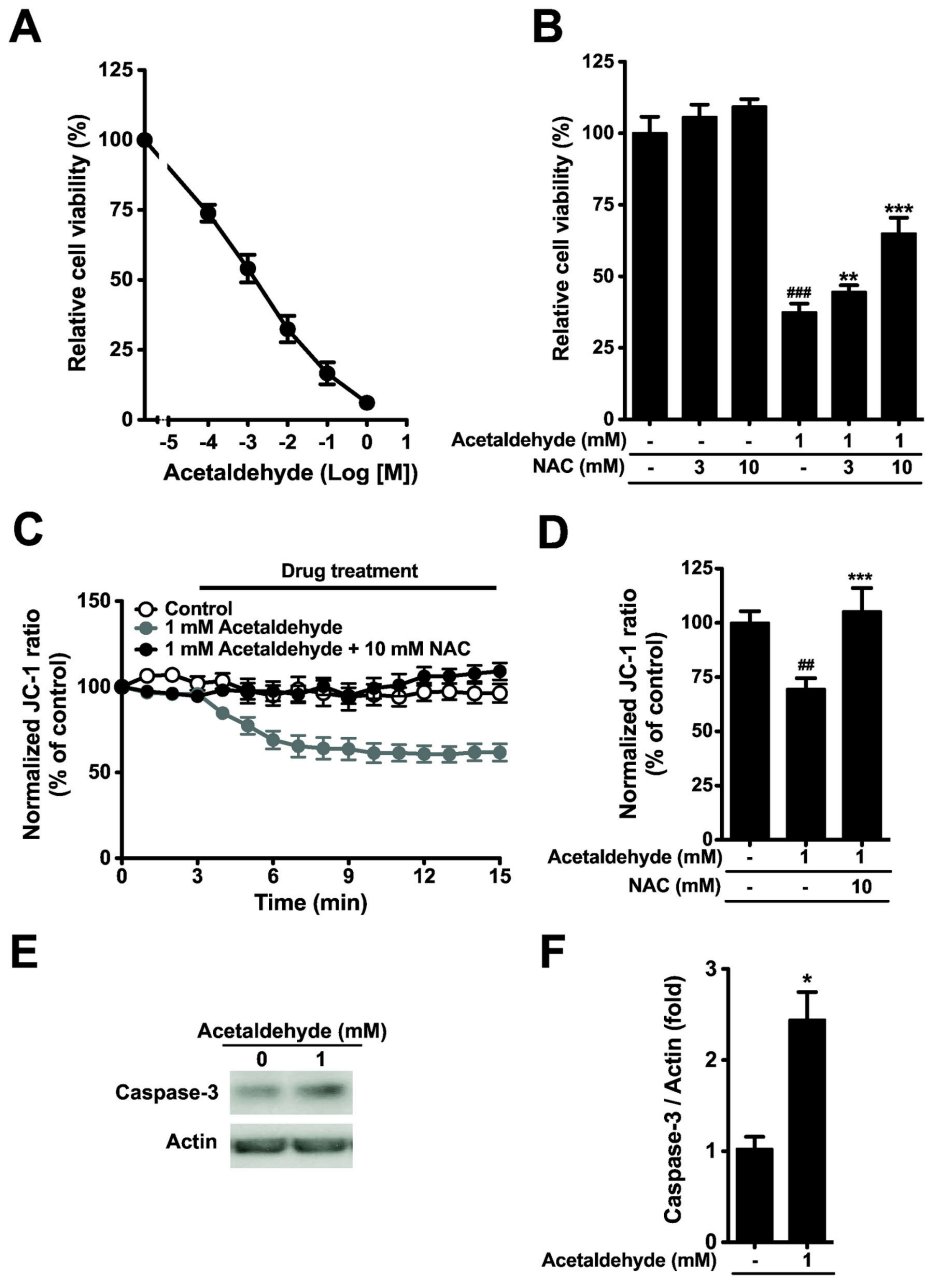
Acetaldehyde induces cell damage that is inhibited by NAC. (A) PDEC were treated with indicated concentrations of acetaldehyde for 4 h, and then cell viability was measured. (B) PDEC were treated with 1 mM acetaldehyde for 4 h with or without 30 min pretreatment of 3 or 10 mM NAC. n = 4 - 13 for each condition, ^###^ *P* < 0.001 compared to control. ** *P* < 0.01 and *** *P* < 0.001 compared to the cells treated acetaldehyde alone. (C and D) MMP measured with JC-1 dye. For the bar graph, MMP was evaluated as averages during drug treatments. n = 5 - 6 for each condition, ^##^ *P* < 0.01 compared to control and *** *P* < 0.001 compared to the cells treated with acetaldehyde alone. (E and F) Caspase-3 activation estimated with Western blot analysis. Cells treated with 1 mM acetaldehyde for 4 h were analyzed to detect activated caspase-3. For the summary bar graph, activated caspase-3 was calibrated by actin level of the samples and presented as relative to untreated control. n = 3 for each condition, *P < 0.05 compared to control.

**Figure 6:**
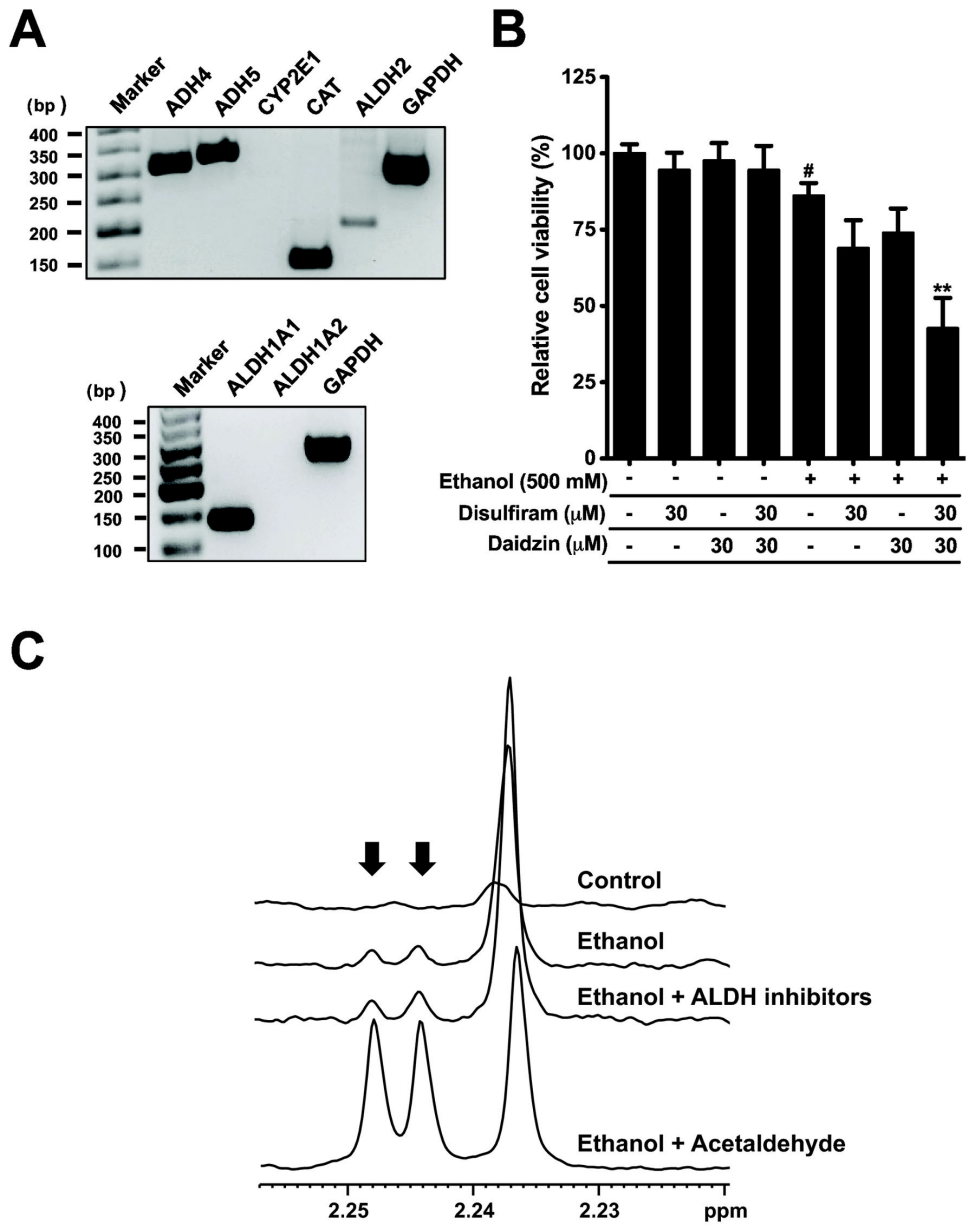
Ethanol is oxidized in PDEC and its metabolite, acetaldehyde, is involved in ethanol-induced cell damage. (A) RT-PCR analysis on expression of oxidative metabolic genes of ethanol in control dog PDEC. Representative gels from at least three independent experiments. (B) Cells were treated with 500 mM ethanol for 4 h with or without 30 min pretreatment with disulfiram (ALDH1 inhibitor), daidzin (ALDH2 inhibitor), or both. n = 6 - 18 for each condition, ^#^ *P* < 0.05 compared to the control. ** *P* < 0.01 compared to the cells treated ethanol alone. Submaximal 500 mM ethanol was used in this experiment to test the further damage by acetaldehyde accumulation. (C) NMR analysis for the detection of cellular acetaldehyde production. Arrows indicate two peaks for acetaldehyde. Estimated acetaldehyde concentrations in the samples are 0, 1.7, 2.1, and 48.4 μM for control cells, cells treated with ethanol, cells treated with ethanol plus ALDH inhibitors, and standard solution including ethanol and acetaldehyde, respectively.

Ethanol is metabolized by oxidative and non-oxidative cellular pathways. One oxidative metabolite, acetaldehyde, is particularly toxic, inducing cell death and malfunction of physiological reactions [35]. To investigate whether acetaldehyde induces cell damage, we first tested the effect of exogenous acetaldehyde using MTS assay (Figure 5A). Acetaldehyde induced damage of dog PDEC at 0.1 to 100 mM, lower concentrations compared to ethanol. Interestingly acetaldehyde-induced cell damage was significantly reduced by NAC as ethanol-induced cell damage (Figure 5B). In parallel, acetaldehyde induced MMP depolarization and caspase-3 activation as ethanol (Figures 5C - F). Again the MMP was restored by NAC cotreatment as we observed with ethanol-induced depolarization (Figure 5D). These data suggest that the oxidative metabolite may mediate ethanol-induced cell damage. We next examined whether dog PDEC express oxidative metabolic enzymes using canine primers available from GenBank (http://www.ncbi.nlm.nih.gov/genbank). RT-PCR assay revealed that alcohol dehydrogenase 4 (ADH4), ADH5, and catalase (CAT), but not cytochrome P450 2E1 (CYP2E1), are highly expressed in our PDEC (Figure 6A). These enzymes help to convert ethanol to acetaldehyde. We also detected expression of acetaldehyde dehydrogenase 1A1 (ALDH1A1) and ALDH2 which convert acetaldehyde to less harmful acetate (Figure 6A). Expression level of these genes related to ethanol oxidation (ADH4, ADH5, and ALDH1A1) was not changed in ethanol treated PDEC for 4 h as determined by Q-PCR (Figure S2). 

Furthermore, we controlled the level of intracellular acetaldehyde using selective ALDH inhibitors to address whether the cytosolic acetaldehyde generated by ethanol is critical for cell damage (Figure 6B). To increase the level of acetaldehyde, PDEC were treated with disulfiram (ALDH1 inhibitor) and daidzin (ALDH2 inhibitor). Each inhibitor alone tends to reduce cell viability but were not statistically significant. With both blockers in combination, cell viability was significantly decreased as expected. We also tested the ADH inhibitors, 4-methylpyrazole (4-MP) and cimetidine to reduce the amount of acetaldehyde. However, they failed to rescue ethanol-induced cell damage significantly (Figure S3). 

Finally, we examined whether acetaldehyde is produced in ethanol-treated PDEC with NMR method. Incubation with 750 mM ethanol increased the characteristic doublet for acetaldehyde at 2.245 ppm (Figure 6C, arrows). The concentration of acetaldehyde in the sample was 1.7 μM. The intracellular concentration could be near or above 100 μM, considering dilution during sampling and evaporation of acetaldehyde during 30 min incubation at 37 °C (see Materials and Methods). Note that acetaldehyde damages the cells significantly at the concentration range (Figure 5A). Acetaldehyde was detected in the cells pretreated with ALDH inhibitors (disulfiram and daidzin) but the amount was marginally increased compared to ethanol alone. 

### Ethanol Increases the Permeability of PDEC Monolayer

**Figure 7:**
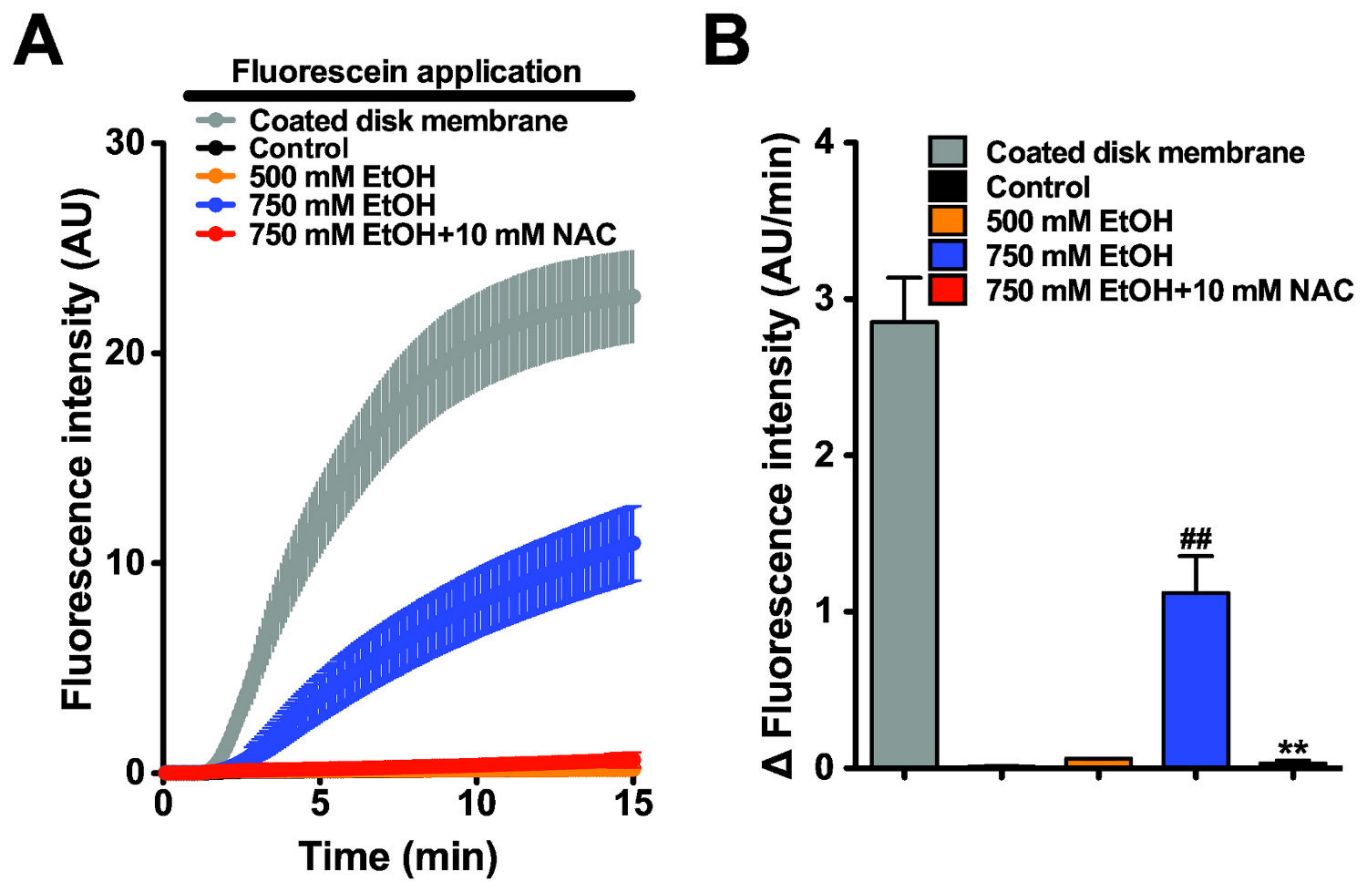
Ethanol increases the leakage of PDEC monolayers. (A) After ethanol treatment of PDEC monolayers for 4 h, their permeability was determined by perfusing fluorescein into the luminal chamber. Coated disk membranes (*N* = 6) without cells represent the maximum permeability, while untreated control monolayers (*N* = 4, overlapped with 500 mM ethanol and therefore invisible) and the monolayers treated with 500 mM ethanol (*N* = 4) had low permeabilities, i.e. tight monolayers. Monolayers treated with 750 mM ethanol (*N* = 6) had a higher permeability to fluorescein. Treatment of PDEC monolayers with 10 mM NAC 30 min before and during 750 mM ethanol (*N* = 3) prevented monolayer leakage. (B) Average rate of fluorescence intensity crossing the monolayers. The rate was estimated from the initial slop between 2 and 8 min. ^##^ *p* < 0.01 compared with the control group. ** *p* < 0.01 compared with 750 mM ethanol-treated monolayers. AU, arbitrary unit. The average and error for control are very small at this scale and therefore invisible.

Ethanol is known to increase permeability of the main pancreatic ductal epithelium [36] and the monolayers of CAPAN-1 pancreatic duct cells [37]. To confirm the ethanol toxicity on PDEC monolayer, we performed a leakage test using fluorescein (Figure 7). Untreated control monolayers were highly tight and there was almost no transfer of the fluorescent dye from the luminal to the serosal sides over the recording period. The initial slope to estimate fluorescein leak was negligible (0.01 ± 0.003 AU/min, black bar in Figure 7B). The monolayers treated with 500 mM ethanol for 4 h did not increase the permeability. In contrast, PDEC monolayers incubated with 750 mM ethanol, the permeability of the dye increased significantly (1.12 ± 0.236 AU/min, blue bar). Interestingly, cotreatment of NAC with 750 mM ethanol removed the damage of PDEC monolayers and the permeability (0.03 ± 0.017 AU/min, red bar on the right) remained same as control monolayers. This protection by NAC parallels the rescue of dissociated single PDEC from cell death.

## Discussion

Due to the clinical relevance, alcohol cytotoxicity has been well studied in a variety of tissues including liver, brain, kidney, and gastrointestinal tract. However ethanol toxicity in the pancreas is less investigated compared to other organs, even though it is one of the principal target organs. Most studies on the effect of ethanol in the pancreas concern pancreatic acinar cells or endocrine β-cells. In acinar cell studies, ethanol not only promotes the premature activation of digestive zymogen [38] but also regulates transcription factors for inflammatory regulation such as nuclear factor κB (NF-κB) and activator protein (AP)-1, inducing pancreatitis [39]. In rats, chronic treatment of ethanol reduced the volume of insulin-secreting β-cells [40] and basal and glucose-stimulated insulin secretion [41]. In addition, ethanol induces β-cell apoptosis *via* mitochondrial dysfunction and oxidative stress [8,42]. For pancreatic ductal epithelial cells, modulation of secretory functions by ethanol has been reported. Ethanol increases permeability of the main pancreatic ductal epithelium [36], and paracellular permeability of monolayers of CAPAN-1 pancreatic ductal cell line [37]. Ethanol also increases secretin-induced fluid secretion by modulating plasma membrane Ca^2+^ channels [43] or increasing intracellular cAMP and Ca^2+^ [44]. The present study focuses on the mechanism of ethanol toxicity in PDEC. 

In this study, we observed significant alcohol toxicity only at ethanol concentration > 500 mM in both single dissociated cells and monolayers incubated with ethanol for 4 h. Apparently these doses are much higher than the possible blood alcohol content after severe drinking (about 85 mM or 0.5%) [26,45] or in excessive alcoholic patients (100 to 200 mM or 0.6 to 1.2%) [46-48]. However, when PDEC were incubated within longer times (48 or 72 h), the ethanol toxicity was detected at lower concentration such as 125 mM (Figure S1A). Comparable ethanol toxicity was observed with primarily cultured pancreatic acinar cells (Figure S1B). In addition, studies from other groups indicate that 170 - 1,700 mM ethanol was needed to cause significant damages of other cell types such as Schwann cells, HeLa, cerebellar granule neurons, PC3, LNCaP, HepG2, HepaRG, and RGM-1 cells [49-55]. Generally lower concentrations were required to damage the cells with longer incubation. Therefore, we assume that repetitive and frequent uptake of ethanol over a prolonged period (e.g. several years) can trigger the same death mechanisms observed under our experimental conditions. 

We have shown that cell death by ethanol is mediated by ROS generation and can be reduced by antioxidant NAC in PDEC (Figures 3A and B). Ethanol decreases the expression of cytosolic antioxidant enzymes including manganese superoxide dismutase, catalase, and glutathione peroxidase in pancreatic tissue from chronic pancreatitis and pancreatic cancer [56]. In contrast, antioxidant NAC decreases oxidative stress by elevating intracellular levels of the endogenous antioxidant glutathione and by augmenting the activities of glutathione peroxidase and glutathione reductase in neuron and hepatocytes [17,57]. These findings support the hypothesis that ROS triggers downstream cell-death mechanisms and therefore reduction of ROS generation by NAC is protective for the ethanol-treated cells. It is unclear how ROS induces mitochondrial membrane depolarization. It could be related to impaired electron transport chain of mitochondria through ROS-induced oxidative stresses [58]. 

In agreement with previous reports [8,12,14], depolarized MMP in PDEC leads to the activation of caspase-3, an initial caspase in apoptosis. It has been shown in several studies that caspase-3 is activated by cytochrome C release from the mitochondria due to MMP depolarization [1,2,59]. Similar to other cell types, ethanol induces mainly apoptosis of PDEC (Figures 1A and 2) after 4 h incubation in ethanol. We observed necrotic cell death only with longer exposure (up to 24 h) to > 500 mM ethanol (Figure 1B). 

After uptake, ethanol is processed in several tissues including liver, brain, and stomach through both oxidative and non-oxidative pathways to reduce toxicity. Previous studies have demonstrated that pancreatic tissues also have the capacity to metabolize ethanol using FAEE synthase and ADH enzymes [39]. In acinar cells, both oxidative and non-oxidative products are toxic [23,26]. It has been shown that the pancreas expresses ADH1, ADH5, ALDH1A1, and ALDH2 proteins [60]. To date, there is no direct evidence that pancreatic ducts can metabolize ethanol. This study indicates that PDEC express metabolic genes capable of ethanol oxidation, such as ADH4, ADH5, catalase, ALDH1A1, and ALDH2 (Figure 6A) and generate acetaldehyde upon ethanol treatment (Figure 6C). Furthermore, pharmacological studies also suggest that ethanol is metabolized in PDEC. Inhibition of both ALDH1 and ALDH2 with disulfiram [61] and daidzin [62] increased ethanol cytotoxicity, presumably through some accumulation of intracellular acetaldehyde (Figure 6B). However, inhibition of either ALDH1 or ALDH2 did not significantly increase ethanol damage, suggesting the presence of a compensatory mechanism between the two enzymes. In contrast, ADH inhibitors, 4-MP [7] and cimetidine [63], did not reduce ethanol-induced cell damage (Figure S3). This result may be due to different ADH isoforms expressed in PDEC (ADH4/5, Figure 6A) and the liver (ADH1/2). A previous study suggests that pancreatic ADH is not sensitive to 4-MP unlike liver ADH [39]. Involvement of acetaldehyde in ethanol toxicity is further supported by shared depolarization of MMP and activation of caspase-3 (Figures 5C - F). Together with the high sensitivity of PDEC to acetaldehyde, these results support the hypothesis that ethanol metabolites also are responsible for ethanol-induced cell damage in PDEC. The exact contribution of ethanol and its metabolites to cell death warrants further detailed studies. 

Finally, we confirmed that ethanol also increases the permeability of PDEC monolayers (Figure 7) consistent with previous reports [36,37]. The ethanol-induced leakage can occur through loss of tight junctions and/or increase of cell death. Maintenance of low paracrine permeability is critical for both bicarbonate secretion into the lumen and confining digestive enzymes inside the ducts during their transport from acinar cells toward the intestine [28,37,64]. Leakage of digestive enzymes to the intersitium is suggested to generate self-digestion and pathological inflammation of pancreatic tissues [37,65]. Therefore ethanol-induced ductal leakage may contribute to the development of pancreatitis.

**Figure 8:**
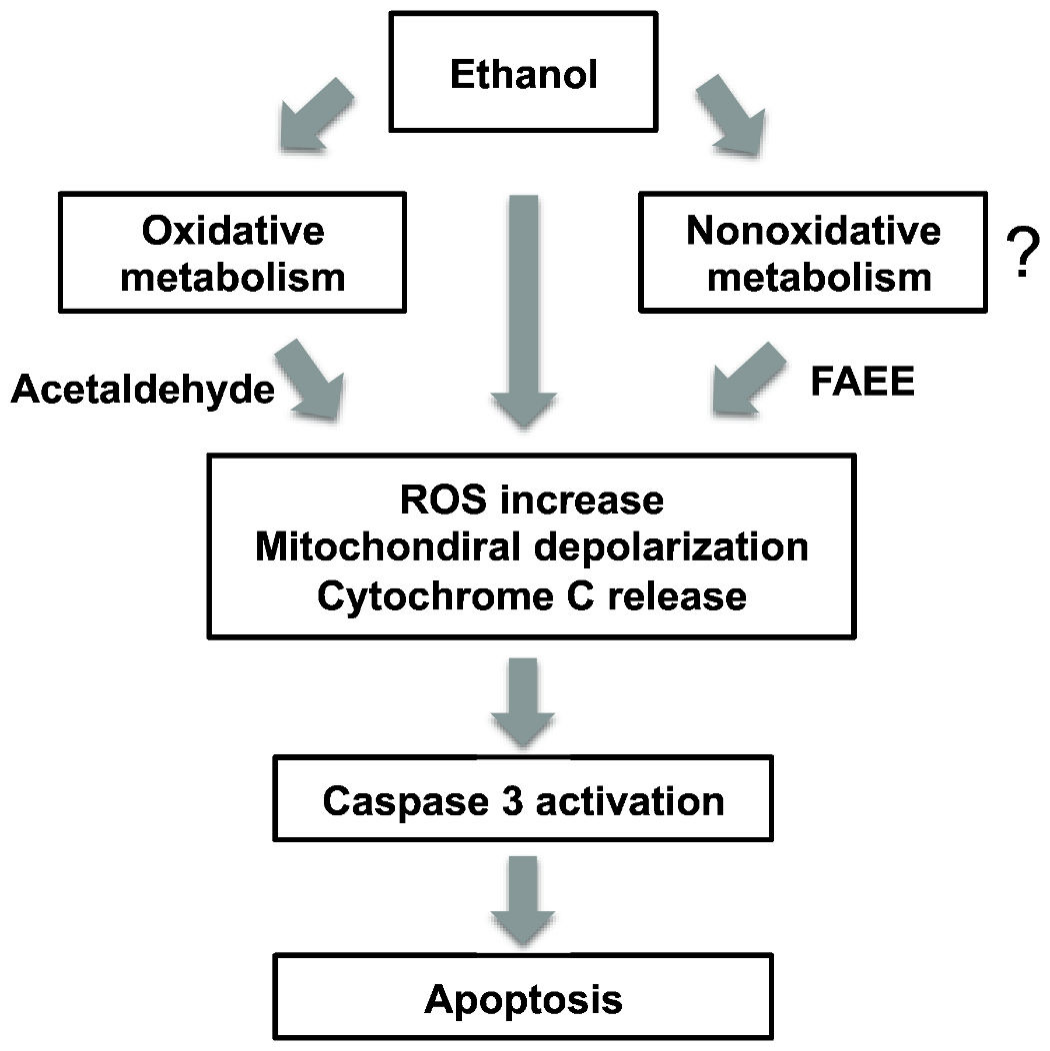
A schematic model for ethanol-induced apoptosis in PDEC. Ethanol can be metabolized through oxidative and nonoxidative pathways. Ethanol and its metabolites (i.e. acetaldehyde and FAEE, fatty acid ethyl ester) induce ROS generation, mitochondrial depolarization, and cytochrome C release that finally activates caspase-3 enzyme to trigger apoptosis.

 In summary, our data presented here for the first time characterize ethanol-induced apoptosis in PDEC *via* generation of ROS, depolarization of mitochondria, and activation of caspase-3 enzyme (Figure 8) and alcohol toxicity on PDEC monolayer. Death of PDEC *via* apoptosis rather than necrosis would be a self-defense mechanism to reduce inflammatory response and pancreatitis. 

## Supporting Information

Figure S1 **Effect of prolonged incubation of ethanol on pancreatic duct epithelial and acinar cells.** (A) PDEC were treated with the indicated ethanol concentrations for 48 or 72 h (*n* = 10 for each condition). The media including ethanol were changed every day considering possible evaporation of ethanol. The trace for 4 h (dotted line) is from Figure 1A for comparison. (B) Primary pancreatic acinar cells were treated with the indicated ethanol concentrations for 4 or 72 h (*n* = 5 for each condition). For 72 h ethanol treatment, the media including ethanol were changed every day. After the treatment, cell viability was determined with MTS assay. The values were calculated relative to the control group.
(EPS)

Figure S2 **Effect of ethanol on expression of oxidative metabolic genes in PDEC.** After incubation of cells with or without 750 mM ethanol for 4 h, total RNA was isolated, and three genes involved in oxidative alcohol metabolism were analyzed with Q-PCR (*n* = 3). Their mRNA levels were presented as relative to ADH4 of control cells. ** *p* < 0.01 and *** *p* < 0.001 compared to ADH4 of control cells. The message levels for each gene did not change before and after ethanol treatment.
(EPS)

Figure S3 **Effect of ADH inhibitors on ethanol toxicity in PDEC.** Cells were treated with 750 mM ethanol for 4 h with or without 30 min pretreatment with ADH inhibitors, 4-methylpyrazole (4-MP), cimetidine, or both. n = 6 - 18 for each condition, ^###^ *P* < 0.001 compared to the control.
(EPS)
